# Channel-assisted minimally invasive repair of acute Achilles tendon rupture

**DOI:** 10.1186/s13018-015-0310-9

**Published:** 2015-10-26

**Authors:** Hua Chen, Xinran Ji, Qun Zhang, Xiangdang Liang, Peifu Tang

**Affiliations:** The Department of Orthopaedic Surgery, The General Hospital of People’s Liberation Army (301 Hospital), 28 Fuxing Road, Wukesong, Beijing, 100000 China

## Abstract

**Background:**

Percutaneous (minimally invasive) suturing is a promising option for Achilles tendon (AT) repair with low rerupture and infection rates. Sural nerve lesions are the major problem to avoid with the technique. A new device was therefore designed for suturing the AT, resulting in channel-assisted minimally invasive repair (CAMIR). The purpose of this study was to compare the clinical and functional outcomes of CAMIR with traditional open techniques.

**Method:**

Eighty two patients with AT rupture were included: 41 for CAMIR, 41 for open repair. All patients followed a standardized rehabilitation protocol. Follow-ups were at 12 and 24 months after surgery. Functional evaluation was based on the clinical American Orthopaedic Foot & Ankle Society score associated with neurologic deficit (sural nerve), calf circumference, range of motion (ROM), and isometric testing.

**Results:**

There was no difference between groups regarding plantar flexor strength, ankle ROM, or calf circumference. CAMIR significantly decreased the operative time compared to open repair (17 vs. 56 min, *P* < 0.0001). Mean scar length was greater in the open repair group (10 vs. 2 cm, *P* < 0.0001). There were no wound complications in the CAMIR group but four in the open repair group (*P* < 0.0001). No deep vein thrombosis, rerupture, or sural nerve injury occurred.

**Conclusion:**

CAMIR and open repair yielded essentially identical clinical and functional outcomes. Sural nerve injuries can be minimized using CAMIR by carefully placing the suture channel with a stab incision and special trocar based on a modified Bunnell suture technique.

## Introduction

Repair of Achilles tendon (AT) ruptures include conservative management using a short-leg resting cast or brace in an equinus position as well as percutaneous, minimally invasive surgery and open sutures with or without augmentation [1, 2]. The best option is still controversial [3]. Some surgeons advocate operative repair because open treatment can ensure tendon approximation and has a lower rerupture rate [4]. However, it is associated with a higher complication rate, including wound infections, skin tethering, sural nerve lesions, and hypertrophic scars, which have caused anxiety for both doctors and patients [2]. Therefore, a percutaneous, minimally invasive suture technique has been developed, decreasing the risk of these complications. Especially, the Achillon suture system has been widely used for minimally invasive suturing of AT ruptures [5, 6].

**Fig. 1:**
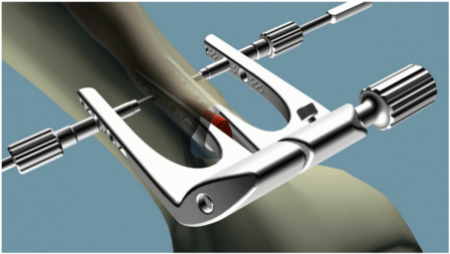
Channel-assisted minimally invasive repair system

The major problem with a percutaneous, minimally invasive technique is sural nerve involvement [7–9]. Some techniques have described measures taken to avoid the risk of nerve injury, such as a modified Achillon technique with the help of an arthroscopic probe [10], endoscopy-assisted percutaneous repair [11], an internal splinting technique [12], and the Mayo needle technique [13–15]. We hypothesized that a channel-assisted minimally invasive repair (CAMIR) that we designed could minimize the possibility of sural nerve injury. The purpose of this study was to compare the clinical and functional outcomes of CAMIR (Fig. 1) with traditional open techniques.

## Methods

This study was devised by a professor of Orthopedics Hua Chen from January 2011 to December 2013 in the Orthopedics Department at General Hospital of People’s Liberation Army (301 Hospital) in Beijing. This study was conducted with the approval of the institutional review board of the 301 Hospital. Written informed consent was obtained from each patient prior to the study.

A sample-size estimation was based on what was needed to detect the difference in complications in the groups. We estimated that more than 30 patients in each group were enough to detect a 20 % difference in American Orthopaedic Foot and Ankle Society (AOFAS) score between groups, with the alpha set at 0.05 and beta at 0.1. An additional 10 % of total participants was planned for each group to make up for possible loss.

### Patients

**Fig. 2:**
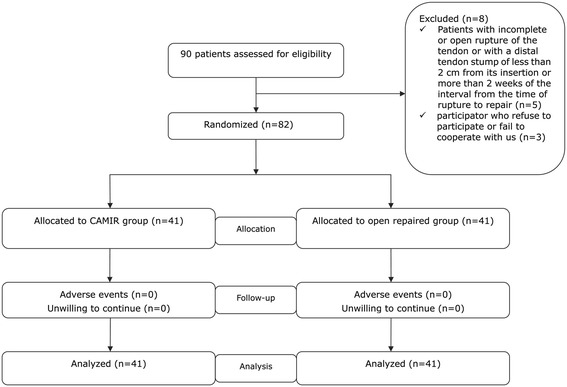
Flowchart of enrolled patients

In all, 90 patients who had suffered an AT rupture because of a sport injury were included in this retrospective study. Of those 90 patients with AT rupture Fig. 2, 41 patients accepted channel-assisted minimally invasive, repair and the rest of patients accepted open repair. The patient was included if he or she had a demonstrable, palpable gap between the ruptured ends, a positive Thompson test, and a distal stump more than 2 cm from the insertion confirmed by ultrasonography. We excluded patients with an incomplete or open rupture of the tendon, a distal tendon stump less than 2 cm from its insertion, a more than 2-week interval from the time of rupture to repair, or comorbidities which could impact clinical outcomes such as diabetes. Any participants who later refused to participate or who failed to cooperate with us in this trial were also excluded.

### Operative technique

Our operative technique entailed a tourniquet applied to the thigh, so the involved limb was exsanguinated in both groups Patients were positioned prone with both legs draped to determine the tension of the ruptured AT after repair and compared it with that on the contralateral side. A single prophylactic 1-g dose of intravenous cephalosporin was administered upon induction. Epidural anesthesia was used in all procedures.

### Open repair group

**Fig. 3:**
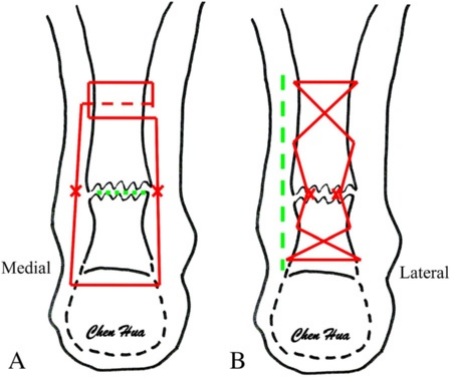
Repair. **a** CAMIR Bunnell suture with the knots outside the tendon. **b** Open Bunnell suture with knots between the tendon ends. *Green dotted lines* indicate placement of the incision. *Red lines* indicate the sutures holding both parts of the rupture

Open repair was performed using an 8- to 12-cm cutaneous medial incision above the rupture (Fig. 3). An end-to-end Bunnell suture was applied using No. 2 Ethibond (Ethicon Inc, Somerville, NJ, USA) sutures augmented with intermittent sutures of absorbable Vicryl 3-0. The paratenon was sutured with absorbable Vicryl 3-0 and the cutis with 2-0 silk interrupted sutures.

### CAMIR group

**Fig. 4:**
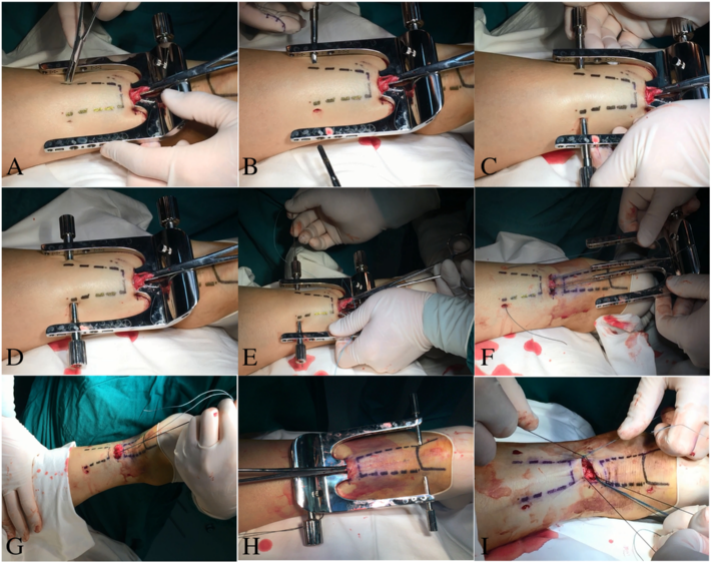
Achilles tendon repair with the help of the CAMIR device. **a** Stab incisions (5 mm) were made through the targeting hole in the limbs. **b** Two trocars with a channel sleeve were introduced through the limb hole, stab incision, and sheath to touch the tendon. There was a two-sided 1.5-cm long blade and blunt tip at the end of the trocar. **c** The paratenon sheath could be cut open longitudinally for about 1 cm with the blade by pushing the system proximally or pulling it distally. The sleeve was then placed through the skin and sheath into the hole in the internal limb. **d** After removing the trocar, the suture channel could move proximally or distally along with the surface of the Achilles tendon, allowing Bunnell suturing. **e** Holding the stump and pushing the device proximally, a No. 2 Ethibond suture was passed through the channel and the tendon with the needle and neutral guide. It exited from the opposite channel. Pulling the device distally, the suture passed through the opposite channel and tendon with the needle and eccentric guide and exited from the channel. The other end of the suture passed through the channel and tendon with the needle and neutral guide and exited from the opposite channel. **f** The channel sleeve was removed, and the device was slowly withdrawn and progressively closed. Thus, the suture exited the incision, trapping the proximal tendon stump in a Bunnell fashion inside the paratenon sheath. **g** After placing sutures in the proximal part of the rupture, we tested the strength of the sutures in the tendon by pulling on the sutures with force. **h** A similar maneuver was performed in the distal stump, with the suture passed through the calcaneus bone tunnel. **i** The two sutures were knotted with the foot in equinus position augmented with intermittent absorbable Vicryl 3-0 sutures

A 1.5-cm transverse incision was made over the palpable gap of the ruptured AT, and the paratenon was exposed without damaging the sural nerve on the lateral side of the incision (Fig. 4). The paratenon sheath was then incised and the tendon stumps identified. Pulling the proximal tendon stump with Kocher forceps, the inner two limbs were placed under the sheath with the stump between the two internal limbs and the outer limbs and outside the skin. Stab incisions (5 mm) were made through the targeting hole in the limbs of CAMIR. The paratenon sheath could be cut open longitudinally for about 1 cm with the blade by pushing the system proximally or pulling it distally. The sleeve was then placed through the skin and sheath into the hole in the internal limb. With the help of CAMIR, the ruptured AT was sutured in Bunnell fashion with a No. 2 Ethibond suture, and then augmented with intermittent sutures of absorbable Vicryl 3-0. The paratenon was then sutured with absorbable Vicryl 3-0 and the cutis with 2-0 silk interrupted sutures. The use of a tourniquet was needed for 20 min in the CAMIR group.

### Postoperative care and rehabilitation

Postoperative care for both groups was the same. After wound closure, a dorsal splint maintained the ankle at 25 to 30° plantar flexion. All stitches were removed 14 days after surgery. A walker boot was given to maintain the ankle at the same flexion degree for another 4 weeks. The patients were told to remove the boot twice a day and perform active dorsiflexion exercises until neutral flexion was achieved. All patients were told to avoid active plantar flexion. At the time of week 6, the walking boot was removed, and active plantar flexion exercises were started, and normal shoes with a 2-cm heel support were permitted. The patients were encouraged to wear normal shoes at 8 to 9 weeks, and active resistive and stretching exercises were started.

### Functional outcome assessment

The follow-up was conducted at 12 and 24 months after surgery. Functional evaluation was based on the clinical AOFAS score along with other findings, such as the length of the scar, neurologic deficit (sural nerve), calf circumference, and range of motion (ROM) of the ankle. At the same time, we employed the hand-held dynamometer to assess the final isometric peak force of plantar flexion of the ankle. The patients were instructed to apply maximum plantar flexion pressure onto the dynamometer sensor (KinCom; produced by Chattecx, Harrison, TN), during which the applied force (in pounds) was measured. This measurement was relatively more reliable in comparison to other methods [6, 13, 16]. The same physiotherapist performed these measurements to eliminate interobserver error. Whether there was no deep vein thrombosis and sural nerve injury was based on color doppler ultrasound and electromyography.

### Statistical methods

The significance threshold was defined as 0.05. Continuous variables, shown as the mean and standard deviation (SD), were compared by the Student *t* test to detect the between-group difference in AOFAS rating score, the length of the scar, neurologic deficit (sural nerve), calf circumferences, final isometric peak force of plantar flexion of the ankle, and the ankle ROM. Qualitative data (sex ratio) between groups was compared by the *χ*^2^ test. All of these statistics were normally distributed. Statistics analysis was performed by SAS Statistical Software 9.1.3.

## Results

From January 2011 to December 2013, a total of 90 subjects participated in this study. Baseline information and demographic characteristics are detailed in Table 1.

**Table 1:** Basic information for patients included in this study

Variable	Open group (*N* = 41)	CAMIR group (*N* = 41)	*P* value
Age (years)	40.3 (10.1)	39.5 (9.2)	0.7087
Sex ratio (M/F)	27/14	29/12	0.8124
Achilles tendon (left/right)	17/24	19/22	0.8239
The length of the distal stump from the insertion (cm)	4.3 (2.7)	4.6 (2.6)	0.6097

Values in parentheses are the SD. Student *t* and *χ* ^2^ tests were used for all statistical analyses

### Clinical evaluation

There was no insignificant difference in calf circumferences, range of motion, isometric peak force of plantar flexion, and AOFAS scores between open repair and CAMIR groups at 12 and 24 months after surgery. However, we see a significant reduction in mean surgical time and length of scar in CAMIR groups in comparison to open repair groups (Table 2).

**Table 2:** Clinical results in this study

Variable	Open group	CAMIR group	*P* value
Mean surgical time (min)	56 (15.8)	17 (4.4)	<0.0001*
Length of scar (cm)	2.0 (0.5)	10.0 (2.5)	<0.0001*
At 6 months after surgery			
Calf circumferences (cm)	30.4 (4.3)	30.3 (4.2)	0.9154
Range of motion	45.2 (5.4)	46.2 (6.2)	0.4384
Isometric peak force of plantar flexion (Ibs)	75.0 (25.9)	79.1 (34.1)	0.5416
AOFAS	82.1 (5.6)	84.2 (6.3)	0.1146
At 12 months after surgery			
Calf circumferences (cm)	32.9 (3.8)	31.9 (3.6)	0.2248
Range of motion	55.4 (4.2)	54.3 (5.1)	0.2896
Isometric peak force of plantar flexion (IBS)	79.6 (28.9)	74.8 (30.8)	0.4689
AOFAS	88.2 (5.6)	90.5 (6.3)	0.0844

Values in parentheses are SD. Student’s *t* and *χ* ^2^ were used for all statistical analyses

*Statistically significant difference between groups

### Complications

No deep vein thrombosis, rerupture, or sural nerve injury occurred in either group. However, four patients in the open repair group experienced delayed wound healing that resolved in about 30 days (*P* < 0.001).

## Discussion

Compared with open repair, percutaneous techniques have reduced the occurrence of major complications, including those that are wound-related [3, 17]. They are also associated with lower rerupture rates when compared with open techniques [16, 18]. The percutaneous techniques allow reapproximation of the tendon ends and better preserve the vascularization of the paratenon and its gliding surface. One of the most popular minimally invasive techniques is with the Achillon device [5, 19, 20], which allows direct visualization of the two ends of the tendon and placement of a transtendinous suture under the paratenon. With our CAMIR technique, the tendon ends can easily be examined and are verified as apposed through the small incision.

The CAMIR technique decreased the potential for sural nerve lesions. In this study, no sural nerve injuries occurred in the CAMIR group. It was known that sural nerve injury is the major problem during percutaneous or minimally invasive suturing of the AT. The first percutaneous suture technique based on Bunnell sutures was that described by Ma and Griffith in 1977 [16]. It caused iatrogenic sural nerve injury, however, with Klein reporting a 13 % rate of these lesions [21]. Nevertheless, it is still commonly used. In the study by Haji [22], 38 patients underwent repair of AT rupture using a modified Ma and Griffith percutaneous repair with transient sural nerve lesions (10.5 %). The Achillon technique is a new tool for percutaneous suture based on box sutures. Although there have been no clinical reports that it produces sural nerve lesions, we believe that there is an inherent risk to the sural nerve with each blind pass of the needle through the skin with the Achillon device. Aibinder’s cadaveric study showed a 14.8 % (8/54) risk of sural nerve violation with each needle pass in neutral position [7]. It is possible that the nerve can be injured during the direct puncture when the needle penetrates the limbs of the Achillon and the suture is pulled through the nerve during withdrawal of the device. The Mayo needle (BL059N, B00 round point spring eye; B Braun Aesculap, Tuttlingen, Germany) is another tool used with the Bunnell technique [2, 14, 19]. It is passed through a stab paratendinous incision and emerges through a central stab incision at the rupture site. The large radius of the curvature of the needle means that the stab incisions tend to avoid the path of the sural nerve. Its disadvantage is that it might produce tethering of the fascia cruris to the tendon. Also, there is a risk of nerve damage because of the blind puncture. However, the technique used in the present study might avoid sural nerve injury by creating a channel for the suture between the skin and tendon with a small skin-stab incision, special trocar placement, and a paratenon cut.

The greater strength produced by this AT repair technique made it possible to accelerate the rehabilitation protocol. The configuration of the Achillon system is new, just like the box suture. Biomechanical studies showed that the Achillon-like configuration had a biomechanical performance similar to that of the Bunnell suture method, the modified Kessler suture, and the Krackow suture, which are widely used for open repairs [23, 24]. However, another cyclic loading laboratory study suggested a different result [25]. Gap resistance was significantly less for the Achillon-like suture (5 cycles) than for the Krackow repairs (502 cycles). All Achillon-like sutures failed during 100-N cycling (102 ± 135 cycles), whereas the Krackow repairs failed during the 190-N cycles (total cycles to failure: 1268 ± 345). Also, augmented Krackow repairs were intact (no gapping) after the 190-N cycles. Our CAMIR technique in this study was based on the Bunnell suture method, which was different from the Achillon technique.

There were no wound problems in this minimally invasive repair group, which may be related to less injury to the paratenon. The paratenon can prevent superficial infection spreading into the deep layers as it is located between the tendon and the skin. It also provides a valuable blood supply to the repaired tendon and avoids skin tethering to the AT. A 1.5-cm transverse incision was made (a small wound) in the paratenon, providing limited exposure of the paratenon. The technique also allows compact closure of the paratenon. All of these conditions protect the blood supply to the AT and promote tissue healing. Another reason for the lack of wound problems is the operative time. In this study, the mean time from skin incision to skin closure was 17.0 ± 4.4 min with the CAMIR technique and 56.0 ± 15.8 min for the open repair. The final reason might be the position of the suture knots. The suture knots are located outside the repaired tendon, which decreases destruction of the blood supply to the tendon.

Rerupture and skin tethering did not occur in this study. This might be related to direct visualization of the tendon stump and augmentation with intermittent sutures after tendon repair, allowing the tendon ends to touch each other completely. The small incision of the paratenon was closed tightly, thereby avoiding skin tethering to the tendon. A significant decrease in the length of the scar contributed to the cosmetic appearance.

Although we achieved satisfactory and good clinical outcomes, our study design and patient numbers were not large enough to provide a statistically valid conclusion. We believe a multicenter, randomized, controlled study of the various methods for AT repair should be conducted in the future. It would then be possible to develop criteria or guidelines for choosing the most advantageous operative strategies for managing the ruptured AT. In addition, we lacked biomechanical data for the modified Bunnell, modified Kessler, Krackow, and box suture techniques. These disadvantages open the door for future study.

## Conclusion

Channel-assisted minimally invasive repair yielded clinical and functional outcomes that were essentially identical to those achieved with open repair—but with a lower incidence of wound complications. The potential of causing sural nerve injury can be minimized using the new suture tool by carefully placing the suture channel with a stab incision and a special trocar based on the modified Bunnell suture technique.
