# Erratum to: Channel-assisted minimally invasive repair of acute Achilles tendon rupture

**DOI:** 10.1186/s13018-016-0347-4

**Published:** 2016-01-20

**Authors:** Hua Chen, Xinran Ji, Qun Zhang, Xiangdang Liang, Peifu Tang

**Affiliations:** The Department of Orthopaedic Surgery, The General Hospital of People’s Liberation Army (301 Hospital), Beijing, China

Unfortunately, the original version of this article [1] contained errors. Firstly, in Table 2, the scar length for the CAMIR group is 2.0(0.5) cm and for open group 10.0(2.5) cm. Secondly, Fig. 1 is cited from a Chinese article [2] with the permission to re-publish in *Journal of Orthopaedic Surgery and Research*. There is an overlap between the two articles. The 30 patients treated by CAMIR in the Chen et al [2], are included in the 41 patients treated by CAMIR in Chen et al [1]. The article [2] is a case report and the data is a retrospective analysis. However, this study [1] is a comparative design, between CAMIR and open repair.
